# Reprogramming: A Preventive Strategy in Hypertension Focusing on the Kidney

**DOI:** 10.3390/ijms17010023

**Published:** 2015-12-25

**Authors:** You-Lin Tain, Jaap A. Joles

**Affiliations:** 1 Departments of Pediatrics, Kaohsiung Chang Gung Memorial Hospital and Chang Gung University College of Medicine, Kaohsiung 833, Taiwan; tainyl@hotmail.com; 2 Institute for Translational Research in Biomedicine, Kaohsiung Chang Gung Memorial Hospital and Chang Gung University College of Medicine, Kaohsiung 833, Taiwan; 3 Department of Nephrology and Hypertension, Laboratory of Renal and Vascular Biology, University Medical Center Utrecht, POB 85500, 3508 GA Utrecht, The Netherlands

**Keywords:** developmental programming, perinatal supplements, nitric oxide, reactive oxygen species, soluble epoxide hydrolase

## Abstract

Adulthood hypertension can be programmed in response to a suboptimal environment in early life. However, developmental plasticity also implies that one can prevent hypertension in adult life by administrating appropriate compounds during early development. We have termed this reprogramming. While the risk of hypertension has been assessed in many mother-child cohorts of human developmental programming, interventions necessary to prove causation and provide a reprogramming strategy are lacking. Since the developing kidney is particularly vulnerable to environmental insults and blood pressure is determined by kidney function, renal programming is considered key in developmental programming of hypertension. Common pathways, whereby both genetic and acquired developmental programming converge into the same phenotype, have been recognized. For instance, the same reprogramming interventions aimed at shifting nitric oxide (NO)-reactive oxygen species (ROS) balance, such as perinatal citrulline or melatonin supplements, can be protective in both genetic and developmentally programmed hypertension. Furthermore, a significantly increased expression of gene *Ephx2* (soluble epoxide hydrolase) was noted in both genetic and acquired animal models of hypertension. Since a suboptimal environment is often multifactorial, such common reprogramming pathways are a practical finding for translation to the clinic. This review provides an overview of potential clinical applications of reprogramming strategies to prevent programmed hypertension. We emphasize the kidney in the following areas: mechanistic insights from human studies and animal models to interpret programmed hypertension; identified risk factors of human programmed hypertension from mother-child cohorts; and the impact of reprogramming strategies on programmed hypertension from animal models. It is critical that the observed effects on developmental reprogramming in animal models are replicated in human studies.

## 1. Introduction

Hypertension remains an important public health challenge and global concern, despite progress in recent years in established therapies. Hypertension may develop early in life; the “thrifty phenotype hypothesis” states that when the fetal environment is deficient in an essential factor or includes a harmful factor, the fetus adapts by favoring growth of crucial organs at the expense of other organs that have a “reserve” capacity and are considered less important for survival under these adverse environmental conditions [1]. The adaptation may cause structural, physiological, and metabolic changes that influence cardiovascular (CV) health later in life. This phenomenon is known as Developmental Plasticity or Programming since the genetic program adapts to existing environmental conditions resulting in different phenotypes [2]. Theoretically, this concept leads to a shift in the therapeutic approach from adult life to early stage, before hypertension is evident. Since the developing kidney is particularly vulnerable to environmental insults and blood pressure is determined by kidney function, renal programming is considered a key player in the developmental programming of hypertension [3].

## 2. Studies in Humans

David Barker and colleagues were the first to acknowledge the association between low birth weight (LBW) and hypertension [4]. Another important observation from the Dutch Hunger Winter Study was that malnutrition during gestation has long-lasting consequences for adult health, including hypertension during stress tests [5]. However, intrauterine development is not the only factor that influences future CV health; although it seems clear that there are critical windows during this period; development during early childhood also plays an important role. Hence, catch-up growth (accelerated postnatal growth) is an important factor for small for gestational age children, as these children have higher systolic blood pressures (BP) than children who grow up normally and are more likely to suffer CV complications in adult life [6]. Therefore, the perinatal period, including intrauterine and early postnatal development, is when programming acts to (re-)set our development.

Numerous epidemiologic studies now support that LBW and prematurity are risk factors for hypertension in later life; importantly, both factors are associated with a low nephron number, although this is not a uniform finding in all ethnic groups [7]. A reduced nephron number leads to higher glomerular capillary pressure and glomerular hyperfiltration, consequently initiating a vicious cycle of rising BP and further nephron loss. Subsequent development of hypertension in people with a low nephron number could result from postnatal activation of the intrarenal renin-angiotensin system (RAS) [8]. Other evidence supporting the link between low nephron numbers and hypertension in humans has come from autopsy kidneys showing that nephron numbers in patients with primary hypertension were 50% lower than that in controls [9]. Although the risk of hypertension has been assessed in a number of mother-child cohorts of human developmental programming research (Table 1), interventions necessary to prove causation and to provide a reprogramming strategy are lacking.

Risks affecting early-life BP of offspring examined in these cohorts include smoking, low vitamin D intake, gestational hypertension, maternal obesity, short-term breastfeeding, excessive postnatal weight gain, and undernutrition [10,11,12,13,14,15,16,17,18,19,20]. Given that multifactorial exposures were studied in a variety of cohorts, it is difficult to compare the relative importance of these risk factors on BP elevation with age. Additionally, almost all current cohort studies involve offspring who have not yet reached middle age and defined cardiovascular endpoints. Hence, these cohorts cannot yet per se establish direct cause-and-effect relationships between specific early-life insults and clinical outcomes; however, may play a role in building an overall picture of the impact of developmental programming in humans.

**Table 1:** Effects of developmental risk on offspring blood pressures in human cohort studies.

Study	Offspring, *n*	Age Range, years	Country	Risk Factors	Reporting Findings Related to BP
Project Viva [10]	746 M/F	3	United States	Maternal smoking	Pregnancy smokers had children with higher SBP
London Health Science Centre [11]	658 M/F	3–16	United Kingdom	High maternal pre-pregnancy BMI, large birth weight	SBP correlates with maternal pre-pregnancy BMI. DBP positively correlates with birth weight, gestational age, and maternal pre-pregnancy BMI
ABCD [12]	1834 M/F	5–6	Netherlands	Low maternal 25-hydroxyvitamin D level (25(OH)D)	↑ 10 nmol/L maternal 25OHD ↓ 0.21 mmHg offspring DBP
Tohoku Study of Child Development [13]	377 M/F	7	Japan	Short-term breastfeeding	BP in the long-term breastfeeding group was significantly lower than in the short-term breastfeeding group
ALSPAC [14]	3062 M/F	9–12	United Kingdom	Pre-eclampsia or gestational hypertension	Offspring of women with pre-eclampsia or gestational hypertension had higher SBP and DBP
ALSPAC [15]	3525 M/F	9.9	United Kingdom	Low maternal 25(OH)D	Maternal 25(OH)D was inversely associated with SBP
ALSPAC [16]	2200 M/F	16	United Kingdom	Excessive gestational weight gain, high maternal pre-pregnancy BMI	Gestational weight gain and maternal pre-pregnancy BMI positively correlate with offspring SBP and DBP
POPS-19 [17]	596 M/F	19	Netherlands	Increased postnatal weight gain	Higher postnatal weight gain, higher SBP
MUSP [18]	2271 M/F	21	Australia	Excessive gestational weight gain	Greater GWG is associated with higher offspring SBP
JPS [19]	1256 M/F	32	Jerusalem	High maternal pre-pregnancy body mass index (BMI)	High maternal pre-pregnancy BMI, high offspring SBP and DBP
Dutch Famine study [20]	359 M/F	59	Netherlands	Undernutrition	SBP and DBP were 2.77 and 1.27 mmHg higher in offspring exposed to famine than those without exposure

Studies tabulated according to offspring age. ABCD = Amsterdam Born Children and their Development; JPS = Jerusalem Perinatal Study; ALSPAC = The Avon Longitudinal Study of Parents and Children; MUSP = Mater-University Study of Pregnancy and its Outcomes; POP-19 = Dutch POPS-19 Collaborative Study Group.

## 3. Studies in Rodents

A vast number of animal studies have documented higher systolic BP in offspring after a variety of perinatal interventions affecting fetal growth and development, including maternal malnutrition in terms of macronutrients and micronutrients, as well as obesity and diabetes. However, a critical assessment of these data show that this phenomenon is mostly observed when external BP (typically tail cuff pressure) is measured but hypertension is not detected in (telemetrically) instrumented animals [21]. This observation suggests that many of these deleterious perinatal conditions affect sympathetic nerve activity. Indeed, a number of recent studies stress the importance of developmental programming of the autonomic nervous system in relation to programmed hypertension [22], and this has been reviewed elsewhere [23].

Developmental plasticity is potentially bidirectional: administering or supplementing beneficial substances or blocking or depleting deleterious substances has demonstrated that hypertension, renal injury and other CV disease symptoms can be ameliorated [24,25,26]. Other groups have studied supplementation of diverse nutrients (glycine, folate, methionine), as reviewed previously [24,27,28]. Most reprogramming strategies encompassed both pregnancy and lactation in order to span the entire period of nephrogenesis in rodents which is roughly from mid-pregnancy to mid-lactation.

Here we summarize studies previously reviewed [24,25,26] and highlight new data documenting reprogramming in both genetic and acquired animal models of hypertension focusing on interventions aimed at vasoactive factors, namely the balance of nitric oxide (NO) and reactive oxygen species (ROS) and the vasodilator epoxyeicosatrienoic acids (EETs). It is quite conceivable that besides hypertension, many other symptoms of CVD could be influenced by such interventions. However, limited information is available on these symptoms and for the sake of brevity, we have restricted this review to hypertension. In the clinic often deleterious factors in the maternal environment are either unknown, difficult to correct or both, therefore, we have primarily organized and restricted our review to pharmacological or well-defined micronutrient reprogramming strategies (indicated as “treatment” in Table 2) [29,30,31,32,33,34,35,36,37,38,39,40,41,42,43,44,45,46,47,48,49] Within each strategy the studies are then grouped by the primary deleterious condition (indicated as “programming mechanism” in Table 2).

**Table 2:** Reprogramming aimed at shifting the NO-ROS balance in developmentally acquired and genetic hypertension models.

Programming Mechanism	Species	Programming Effects	Treatment	Period of Treatment	Reprogramming Effects	Age at which Effects Were Measured	Ref.
50% caloric restriction during pregnancy	Wistar rats	Hypertension ↑ Oxidative stress ↓ EDVD	Vit. C or E	14 to 16 weeks of age	↓ BP ↓ Oxidative stress. Normalized EDVD	14 to 16 weeks of age	[29]
50% caloric restriction during pregnancy	Wistar rats.	↑ BP LBW Endothelial dysfunction Impaired renal function ↓ Glomerular number	Micronutrients mix: Vit. C, E, selenium and folic acid	During pregnancy	↓ BP Prevented LBW ↑ Vascular function	Until 14–16 weeks of age	[30]
Genetic hypertension/Aging	SHR and aging WKY rats	Hypertension Proteinuria	l-arginine plus antioxidants (Vit. C, E and taurine)	2 weeks before until 4 or 8 weeks after birth	↓ BP Prevented proteinuria. Transient ↓ of oxidative stress	Every 6 weeks until 50 weeks of age	[31]
Genetic hypertension.	SHR and WKY rats	Hypertension. High RVR Wide range of RBF autoregulation	l-arginine plus antioxidants (Vit. C, E and taurine)	2 weeks before until 8 weeks after birth	↓ Hypertension and RVR RBF autoregulation shifted towards WKY pattern	At 9 week old	[32]
Genetic hypertension, renal injury	FHH rats	Hypertension Progressive renal injury	l-arginine plus antioxidants (Vit. C, E and taurine)	2 weeks before until 4 weeks after birth	Prevented hypertension. ↓ Proteinuria ↓ Glomerulosclerosis in females	Until 36 weeks of age	[33]
50% caloric restriction during pregnancy and lactation	Sprague-Dawley rats	LBW, impaired renal function, renal injury	Citrulline	During pregnancy and lactation	↑ BP ↓ Renal injury ↑ Nephron number	Until 12 weeks of age	[34]
Genetic hypertension.	SHR and WKY rats	Hypertension. ↓ Renal NO at 2 weeks. ↓ Renal gene expression of ASS and ASL at 2 weeks	Citrulline	2 weeks before until 6 weeks after birth	↓ Hypertension in females and until 20 weeks in males ↑ Renal NO at 2 weeks	Until 50 weeks of age	[35]
Diabetes (STZ) during pregnancy and lactation	Sprague-Dawley rats	Hypertension, renal injury	Citrulline	During pregnancy and lactation	↓ BP ↓ Renal injury	Until 12 weeks of age	[36]
Dexamethasone during pregnancy	Sprague-Dawley rats	Hypertension	Citrulline	During pregnancy and lactation	↓ BP	Until 12 weeks of age	[37]
L-NAME during pregnancy	Sprague-Dawley rats	Hypertension	Citrulline	During pregnancy and lactation	↓ BP ↓ ADMA	Until 12 weeks of age	[38,39]
Genetic hypertension, renal injury	FHH rats	Hypertension Progressive renal injury	Molsidomine (NO donor)	2 weeks before until 4 weeks after birth	Prevented hypertension. ↓ Glomerulosclerosis.	Until 36 and 42 weeks of age	[40]
Genetic hypertension	SHR	Hypertension Proteinuria	Vit. C, E and Molsidomine (NO donor) or Tempol (SOD mimetic)	2 weeks before until 4 or 8 weeks after birth	↓ BP Prevented proteinuria	Every 6 weeks until 50 weeks of age	[41]
Genetic hypertension, renal injury	FHH rats	Hypertension Progressive renal injury	Molsidomine (NO donor)	From 2 weeks before until 4 weeks after birth	Differential regulation of renal ribosome protein genes at 2 days and 2 weeks of age ↓ Renal ribosome structures at 2 weeks of age	Until 42 weeks of age	[42]
Genetic hypertension	SHR	Hypertension	Pentaerythritoltetranitrate (NO donor and antioxidant)	During pregnancy and lactation	↓ BP in females, epigenetic changes relating to eNOS in the aorta	At 6 and 8 months of age	[43]
Dexamethasone during pregnancy	Sprague-Dawley rats	Hypertension	Melatonin	During pregnancy and lactation	↓ BP ↑ Nephron number	Until 16 weeks of age	[44]
Dexamethasone during pregnancy	Sprague-Dawley rats	Hypertension	Melatonin	During pregnancy and lactation	Preserved histone deacetylase gene expression	Until 16 weeks of age	[45]
High fructose intake during pregnancy	Sprague-Dawley rats	Hypertension	Melatonin	During pregnancy and lactation	Prevented hypertension Increased renal NO	Until 12 weeks of age	[46]
Protein restricted diet during pregnancy	Wistar rats	Hypertension Vascular dysfunction Microvascular rarefaction ↑ Oxidative stress	Lazaroid (inhibitor of lipid peroxidation)	During pregnancy	Prevented hypertension. Improved vascular function and microvascular rarefaction. ↓ Oxidative stress.	Until 12 weeks of age	[47]
Genetic hypertension	SHR and WKY rats	Hypertension	Pyrrolidine di-thio-carbamate (NF-κB inhibitor)	2 weeks before until 4 weeks after birth	↓ BP ↑ Natriuresis at 4 weeks ↓ Oxidative stress markers	Until 28 weeks of age	[48]
Genetic hypertension	FHH rats	Hypertension Progressive renal injury	Pyrrolidine di-thio-carbamate (NF-κB inhibitor)	2 weeks before until 4 weeks after birth	Prevented hypertension ↓ Glomerulosclerosis	Until 36 and 42 weeks of age	[49]

## 4. Reprogramming the Balance of Nitric Oxide and Reactive Oxygen Species

Most reprogramming strategies have been directed at influencing the balance of nitric oxide (NO) and reactive oxygen species (ROS). For the sake of clarity, we therefore restrict our discussion mainly to these studies. NO-ROS balance may well play a role at different levels of control: setting of central sympathetic output and baroreceptor setting [50], intrarenal vasomotor sensitivity and sodium chloride handling [51], and peripheral vasomotor sensitivity [52]. Structural effects in terms of nephrogenesis [53] and angiogenesis [52,54,55] are also possible. Thus, the position of the NO-ROS balance in the hierarchy and feed-back of programmed long-term blood pressure control is still unclear. We have focused on interventions during nephrogenesis. Nephrogenesis requires a fully active renin angiotensin system and it is well known that blocking the renin-angiotensin system during nephrogenesis leads to malformed renal arterioles and in the long-term, malignant hypertension, despite the initial lowering of BP [56]. Therefore, even though changes in the intrarenal renin-angiotensin system during developmental programming are well recognized [57], we have not included studies where blocking the renin-angiotensin system after the period of nephrogenesis prevented genetic hypertension in spontaneously hypertensive rats (SHR) [58,59,60] or acquired hypertension after maternal protein or caloric restriction [61,62,63,64]. The practical consequence of these observations is that different drugs (or combinations of drugs) are required to prevent or control hypertension at different stages of life.

The overview of studies in Table 2 illustrates that the same reprogramming interventions aimed at NO-ROS balance, such as perinatal supplements of citrulline [34,35,36,37,38,39] or melatonin [44,45,46], can be protective in both genetic and developmentally programmed hypertension. This may indicate common pathways, whereby both genetic and acquired developmental programming converge into the same phenotype. An alternative strategy is to increase NO availability by the administration of Sildenafil (the well-known phosphodiesterase-5 inhibitor Viagra). This has been shown to ameliorate intrauterine growth retardation (IUGR) in offspring of nutrient-restricted sheep [65]. Even if these pathways and candidates are not primary causal factors, they still suggest that reprogramming strategies could be applicable in a broad range of pro-hypertensive developmental conditions. This would seem a practical finding for translation to the clinic.

## 5. Probing the Renal Transcriptome

Converging pathogenic pathways in the development of hypertension are also suggested by analysis of the transcriptome of developing and adult kidneys of different rodent models of developmentally programmed and genetic hypertension [26,66,67,68,69,70]. Differentially expressed genes (DEGs) are already observed at two and seven days of age (mid-nephrogenesis in the rat) [42,66,69] and two weeks of age [46,66,67,68,69,70], probably before development of the hypertensive phenotype. These DEGs could be regarded as important for programming of hypertension.

Because reduced nephron number plays a crucial role in programmed hypertension [8], we used to analyze a number of nephrogenesis-related genes in offspring kidney at 2 weeks of age in a variety of nutritional programming models [66]. Among them, *Osr1*, *Gdnf*, *Fgf2*, *Gata3*, *Lif*, and *Ret*, were modified above the chosen threshold in the caloric restriction model. In addition, *Fgf10*, *Igf1*, *Six1*, *Grem1*, *Wnt11*, *Fgf2*, *Etv4*, *Osr1*, *Gdnf*, *Spry3*, *Bmp2*, and *Notch3* had significant differential expressionin the maternal diabetes model. Of note is that *Gdnf* encoding glial cell line-derived neurotrophic factor (GDNF), was similarly upregulated in four diverse programming models. Given thatGDNF is essential for the morphogenesis of the ureteric bud during nephrogenesis [71], and that GDNF^+/−^ heterozygous mice develop reduced nephron endowment [72], increased *Gdnf* expression is presumably a compensatory mechanism in response to impaired nephrogenesis. We also observed that *Fgf2* and *Osr1* were present in both caloric restriction and maternal diabetes models, while *Six1* was present in diabetes and high fructose models. Whether these genes are related to reduced nephron endowment in response to different maternal insults leading to programmed hypertension, and in particular whether their levels of expression respond to reprogramming interventions aimed at NO-ROS balance, awaits further elucidation.

The renal transcriptome is profoundly altered in adult offspring in the presence of hypertension [26,42,67,68,69,70]. Although many DEGs in adult rats are probably related to secondary changes in the kidney, it is intriguing that some DEGs that already manifest during renal development persist into adulthood. We found that arachidonic acid metabolism is a significant related Kyoto Encyclopedia of Genes and Genomes (KEGG) pathway in adult offspring kidneys from both the high fructose and prenatal dexamethasone exposure models [46,67]. In addition, arachidonic acid metabolites play important roles in BP control. Thus, we analyzed four upregulated genes in the arachidonic acid metabolism pathway, including *Hpgds*, *Ephx2*, *Ptgds*, and *Ptgs1*. Both high fructose intake and prenatal dexamethasone exposure upregulated gene expression of *Hpgds*, *Ephx2*, *Ptgds*, and *Ptgs1* [66], in agreement with RNA sequencing data. Prenatal dexamethasone exposure leads to high urine angiotensin II excretion at 4 to 8 weeks of age and increases in systolic blood pressure at 8 weeks without a change in plasma or renal angiotensin II levels, suggesting that luminal angiotensin II concentrations may be driving the development of hypertension via increased sodium transport [73]. Subsequently, the same group showed that the hypertensive effect of maternal dietary protein deprivation in male offspring could be prevented by inhibiting glucocorticoid synthesis with the 11 beta-hydroxylase inhibitor metapyrone [74]. Normalization of blood pressure was accompanied by the normalization of nephron number, suggesting that prenatal dexamethasone exposure primarily inhibits nephrogenesis. It is well-known that angiotensin II drives ROS formation [52], but the hierarchy between the angiotensin and arachidonic acid pathways in relation to NO-ROS balance is unclear and has not been explored in developmental programming of hypertension.

## 6. Epigenetics in Programming and Reprogramming

The environment during nephrogenesis is critical for the development of hypertension in adult life. This environment may impact on nephron formation, but also on setting the activity of key processes such as sodium transport and vasomotor function. Because of the high turn-over rate of epithelial and endothelial cells it is likely that that epigenetic processes involving DNA methylation, histone acetylation and transcriptional activation are involved in setting and resetting the activity of these processes over the lifespan of the organism. Particularly in the absence of a change in nephron number it is likely that epigenetic processes will be involved. We studied the prenatal dexamethasone model in rats, where maternal treatment with dexamethasone leads to impaired nephrogenesis and hypertension in the offspring, and found increased histone deacetylase1 in the kidneys accompanied by increased angiotensin II AT1 receptor and the thiazide sensitive transporter NCC expression. Both hypertension and these molecular changes could be prevented by adding l-citrulline to drinking water during pregnancy and lactation. In follow-up studies with the dexamethasone model we again found increases in renal histone deacetylases (HDACs) and decreased expression of genes important for nephrogenesis. These deleterious effects and the resulting hypertension could all be reversed by the antioxidant melatonin [26,45]. Recently, this approach has been taken one step further by addition of melatonin to the culture medium used for assisted reproductive technologies. Rats generated by such technologies become hypertensive. Addition of melatonin to the embryo culture medium prevented this, and increased NO availability by reversing the increased methylation of the endothelial nitric oxide synthase (eNOS) gene promoter and restoring eNOS expression and vasodilator function in resistance arteries [75]. Note that melatonin, besides its antioxidant action, has pleiotropic central and peripheral effects [76].

The importance of enhanced methylation of the eNOS promoter in programming hypertension was also supported by the observation that treating pregnant and lactating SHR with pentaerythritoltetranitrate (PETN) a tolerance-free NO donor, decreased blood pressure of the female offspring and enhanced aortic eNOS expression, likely via distinct epigenetic changes of the promoter and transcriptional activation [43]. Antihypertensive effects were not found in male SHR offspring but this is probably because BP was not measured before six months of age. Our experience with perinatal interventions in SHR is that antihypertensive effects generally wane in male but persist in female offspring [31,35,49,77]. Although these findings in resistance arteries [75] and the aorta (a conduit vessel) [43], support important epigenetic regulation of eNOS in early development, it remains unclear whether these effects can be extrapolated to eNOS in the kidney.

## 7. Reprogramming via Inhibition of Soluble Epoxide Hydrolase

The *Ephx2* gene encodes soluble epoxide hydrolase (SEH), a cytoplasmic enzyme that converts vasodilator epoxyeicosatrienoic acids (EETs) to less active dihydroxyeicosatrienoic acids (DHETs) [78]. In multiple experimental studies, inhibition of SEH has been shown to have antihypertensive effects and reduces target organ injury, leading to current clinical trials [79]. Moreover, SEH gene deletion leads to lower blood pressure than in wild-type mice [80] and protects against deoxycorticosterone acetate (DOCA)-salt and Angiotensin-II-induced hypertension [81,82]. It was well known that expression of *Ephx2* was upregulated from 3 week to 18 weeks age in spontaneously hypertensive rats (SHR) and it could be demonstrated that this was already the case at 2 and 14 days of age, remaining upregulated till nearly one year of age [77]. Administration of dexamethasone to pregnant rats during the last week of their 3-week gestational period is a well-known stimulus to program hypertension and renal injury in the rat [83,84]. We have previously shown that hypertension in the offspring in this model can be prevented by supplements of melatonin and by citrulline during pregnancy and lactation [37,44]. Interestingly, in this acquired model, upregulation of *Ephx2* expression and SEH protein expression and activity was shown [67]. Furthermore, this was also the case in a completely different model of programmed hypertension, namely maternal high-fructose diet [46]. Thus, common pathways, whereby both genetic and acquired developmental programming converge into the same phenotype, also appears in next generation sequencing studies where expression of the same genes, such as soluble epoxide hydrolase, are upregulated in the kidneys [69]. Indeed, we could demonstrate that inhibition of SEH during gestation and lactation in SHR dams with the orally active inhibitor 12-(3-adamantan-1-yl-ureido)-dodecanoic acid (AUDA) could persistently reduce blood pressure up to 24 weeks of age in the offspring, therefore, for at least 20 weeks after stopping exposure to AUDA [77]. Notably, this was accompanied by complex changes in renal epoxide and diol profiles, as well as the overall profile of arachidonic acid metabolites [77], suggesting that BP-lowering effects of perinatal SEH inhibition are not simply explained by persistent reduction in renal SEH activity but rather by more complex and temporally dynamic interactions between the renal SEH, lipoxygenase, and cyclooxygenase pathways. Hence, in the three models of hypertension, two programmed by maternal stress and one genetic, *Ephx2* gene expression and SEH activity seem to play a direct and indirect role. It would be interesting to see whether concomitant pharmacological inhibition of SEH can also ameliorate hypertension in the dexamethasone and high fructose models.

In adult SHR AUDA was ineffective at reducing BP; a phase 2 trial with the SEH inhibitor AR9281 was stopped, probably because of inefficacy in adults with mild hypertension [85]. However, administration in prehypertensive or borderline hypertensive subjects has not been probed, let alone to pregnant women with placental insufficiency resulting in developmental risk factors such as intrauterine growth retardation (IUGR) and possibly long-term hypertension.

## 8. Conclusions

Studies in short-lived animals, with controlled interventions across their early lifespan, have provided strong evidence of a causal relationship for developmental programming. However, this is far more challenging in human studies, which need longer-term follow-up. Furthermore, early-life exposures in humans are much more multifactorial and complex. Nevertheless, several groups have initiated trials with Sildenafil in pregnant women with IUGR [86,87]. The long-term effects on blood pressure in these children will be a key observation.

Patients with prehypertension have an increased risk of complete hypertension, target organ damage, and cardiovascular morbidity and mortality. Extensive experimental animal studies and epidemiological observations have shown that environmental influences during early life elicit programmed hypertension in adult life. Studies with animals employing a reprogramming strategy based on shifting the NO-ROS balance towards NO in order to prevent hypertension have demonstrated converging results in quite different models. Hence, early detection of individuals that are at risk for hypertension and early intervention to reprogram hypertension may well allow us to reduce the future burden of hypertension.
